# Membrane Associated Synaptic Mineralocorticoid and Glucocorticoid Receptors Are Rapid Regulators of Dendritic Spines

**DOI:** 10.3389/fncel.2016.00161

**Published:** 2016-06-21

**Authors:** Manuela F. Russo, Sarah R. Ah Loy, Andrew R. Battle, Luke R. Johnson

**Affiliations:** ^1^Translational Research Institute, Institute of Health and Biomedical Innovation, QUTBrisbane, QLD, Australia; ^2^School of Psychology and Counseling, QUTKelvin Grove, QLD, Australia; ^3^School of Biomedical Sciences, QUT, Gardens PointQLD, Australia; ^4^Department of Psychiatry, Centre for the Study of Traumatic Stress, USUBethesda, MD, USA

**Keywords:** LIM kinase, ADF/cofilin, amygdala, genomic, non-genomic, stress, circadian, fear

Unemployment is never a good situation to be in. For ~25 years the direct membrane acting, non-genomic, variety of the mineralocorticoid receptor (MR) and the glucocorticoid receptor (GR) have been in want of a credible function. Even the existence of a membrane MR (mMR) and membrane GR (mGR) was questioned. Recent data suggest this is changing; mMR and mGR are located in synapses and surrounding space of rapidly plastic dendritic spines (Prager et al., 2010; Komatsuzaki et al., 2012; Yoshiya et al., 2013). Emerging evidence, reviewed below, links mGR activation to rapid changes in dendritic spine size and number. These structural changes can be both experimentally induced and are also naturally induced during circadian fluctuations in corticosterone levels, and are rapidly mediated by kinase signaling. Moreover, mMR activation may reverse these changes. Previous data also links the mMR to receptor trafficking and regulation of synaptic transmission. Collectively mMR and mGR mediated rapid regulation of synaptic structure and function is central for learning and memory. In this paper we briefly summarize the history of the mMR and mGR and then update on the emerging evidence of their reported functions. Here we argue that there is enough evidence to consider these rapidly acting non-genomic membrane receptors as members of the community of fully employed receptors.

While the function of mMR and mGR remains elusive or unknown, the existence of these receptors has historically been well defined by many research groups (for reviews see Prager and Johnson, 2009; Chaouloff and Groc, 2011; Groeneweg et al., 2012; ter Heegde et al., 2015). This opinion paper focuses on the role of mMR and mGR in neurons. It should be noted that the membrane form of the receptor is not exclusive to the brain and has been well-characterized in many cellular systems, including for example, the immune system (Buttgereit et al., 2015). Recent developments in our understanding of corticosteroid signaling highlights the importance of rapid pulsatile ultradian signaling to mMR and mGR, which provides an upstream signaling process well-suited for a rapid membrane response to corticosteroids. These findings, reviewed extensively by Joels et al. (Joëls et al., 2012; Sarabdjitsingh et al., 2012) provide evidence and rationale for the existence of mMR and mGR.

In 2005, we (Johnson et al., 2005) identified GR receptors in the post-synaptic density (PSD) as well as in dendrite spines, dendrites, soma, nuclei, and also pre-synaptic terminal regions and glia processes. In 2010, we identified both GR and MR in the PSD as well as in dendritic spines, dendrites, soma, and pre-synaptic terminal regions (Prager et al., 2010). Importantly, these findings established ultra-structural micro-anatomical evidence for both the membrane and intracellular nature of potential mMR and mGR in the distal regions of neuron architecture. In both of these studies the authors used transmission electron microscopy combined with immunocytochemistry labeled with the electron dense chromogen diaminobenzidine (DAB). Although DAB has a reduced spatial sensitivity (due to the amplification of the antigen signal away from the MR and GR antigen), it has very good tissue permeability and also provides amplification of the antigen signal. Consequently, this approach has enabled identification of MR and GR at novel intracellular and membrane sites. For example, MR was localized to Golgi apparatus, mitochondrial membranes and in the post-synaptic density itself (Johnson et al., 2005; Prager et al., 2010). The disadvantage of the DAB method is its reduced spatial sensitivity due to the amplification of the antigen signal away from the MR and GR antigen. A subsequent study by Kawato and colleagues (Yoshiya et al., 2013) used immunogold techniques with increased spatial sensitivity (and decreased penetration sensitivity) to confirm the findings of Johnson and Prager for synaptic and dendritic spine locus of MR and GR (Johnson et al., 2005; Prager et al., 2010). In summary, micro-anatomical evidence for synaptic associated MR and GR in the distal regions of neuron architecture has been established with different techniques and in different brain regions. These synaptic mMR and mGR can be argued to be established receptors, however their function has been less well established until recently.

A central premise of contemporary neuroendocrinology is the binding of adrenal cortex released corticosterone to MR and GR and their translocation to the nucleus (for review see de Kloet et al., 2008; Prager and Johnson, 2009). It is important to note that the binding of cortisol (humans) and corticosterone (rodents) to MR and GR in the brain is organized around differences in affinity, with MR having a higher affinity and being occupied at basal levels, and GR having a lower affinity and being occupied at circadian peaks and periods of stress (de Kloet et al., 2008; Groeneweg et al., 2012). MR and GR are found throughout the brain, binding of corticosterone, in the brain, was first described by McEwen et al. (1968), who showed that the steroid bound at high concentrations in limbic areas, especially the hippocampus. Importantly, it was noted in this seminal study that its mode of action was likely via binding to the genome, as binding was observed in neuronal nuclei (McEwen et al., 1968). Many subsequent studies have supported the genomic activation of corticosterone theory. Thus, a central premise of neuroendocrinology is the transactivation and transrepression of gene products mediated by corticosterone binding in the nucleus (for review examples see Tasker et al., 2006; de Kloet et al., 2008; Prager and Johnson, 2009; Joëls et al., 2012; ter Heegde et al., 2015). Twenty-three years after the identification of corticosterone binding in the brain, Orchinik et al. (1991) identified that corticosterone also bound to neuronal membranes. The initial identification of corticosterone binding to neuronal membranes, together with the studies of Johnson et al. (2005) and Prager et al. (2010) suggests corticosterone most likely also binds to synaptic membranes. A key role of these synaptic receptors could be the regulation of dendrite spines.

Membrane GR regulates dendrite spine growth and MR plays a regulatory role in dendrite spine structure. Recent pioneering work by Gan and colleagues suggests that endogenous corticosterone levels drive spine structural change (Liston et al., 2013). Working in an *in vivo* model they found that circadian fluctuations in corticosterone drive spine growth and retraction. Circadian cortisol peak is associated with GR dependent spine growth, with spine pruning occurring in the circadian nadir. The influential relationship that MR and GR have on spine formation during circadian glucocorticoid peaks and troughs indicates that they likely have important implications for memory retention and learning. In a learned motor skill task Liston et al. (2013) found an increase in spine formation in mice trained during circadian peaks, which reinforced learning and strengthened long-term memory retention. Strong evidence for the non-genomic nature of these GR mediated effects comes from the association of these effects with LIM kinase-1 phosphorylation. Just 20 min after direct application of corticosterone to the cortex increases in LIM kinase-1 phosphorylation are detected, moreover GR spine changes are not detected in LIM1 knockout (*LIM1*^−∕−^) mice. These *in vivo* studies on cortical neuron spines are supporting a growing body of evidence of non-genomic mediated corticosterone regulation of spine structure. Furthermore, an earlier study by the same group (Liston and Gan, 2011) also showed the regulatory role of glucocorticoids in dendritic spine development and plasticity *in vivo*. Addition of both the GR selective antagonist mifepristone and the MR antagonist spironolactone (both separately and co-administered) in the developing cortex disrupted spine formation, indicating that both receptors play roles in spine development. Conversely, extended corticosterone interaction led to elimination of both older and younger spines. Sleep has also been shown (Yang et al., 2014) to promote the formation of dendritic spines. Examining both non- and sleep-deprived (SD) mice, the authors found that spine formation was reduced significantly in SD mice compared to the non-SD mice. In summary, circadian fluctuations of corticosterone likely regulate spine structure via mMR and mGR.

*In vivo* studies have also identified the rapid regulation of dendrite spines by GR and the apparent cyclic and reversible nature. On examination of both hippocampal CA1 dendrite spines and CA3 dendrite thorns, Komatsuzaki et al. (2012) and Yoshiya et al. (2013) found the density of spines had increased after 1 h post-corticosterone administration. However by 5 h of corticosterone application these increases were reversed. Like the effect on CA1 spines corticosterone also increased the density of CA3 thorns within 1 h, also in a dose dependant manner. The corticosterone mediated increases in spines and thorns were both dependant on GR and on intracellular signaling including phosphorylation of ERK/MAPK. Furthermore, the rapid corticosterone mediated increases in spine density was also dependent on glutamatergic transmission with NMDA and AMPA receptors also inhibiting the corticosterone mediated increases in spine density. Thus, dendrite spine and thorn density is rapidly increased by corticosterone via glucocorticoid, NMDA and AMPA receptors; and the phosphorylation of intracellular signaling kinases. Given that mGR are observed at the PSD (Komatsuzaki et al., 2012; Yoshiya et al., 2013), membrane associated GR are positioned to interact with NMDA and AMPA receptors and the ERK/MAP and LIM kinase pathways to drive rapid changes in spine number and size.

Corticosterone regulates both an increase and decrease in dendrite spine density that is mediated by both GR and MR. In both *in vivo* studies (Gan and colleagues) and *in vitro* studies (Kawato and colleagues) the rapid increase in spine density mediated by GR activation was found to be reversible and cyclic. Kawato and colleagues observed that application of corticosterone increased spine density at 1 h which then decreased back to the control levels at 2 and 5 h (Komatsuzaki et al., 2012). Furthermore, spine increases in GR dependent spine densities are reversed during the circadian nadir (Liston et al., 2013). Gan and colleagues also found that excess GR also reduced spine density (Liston et al., 2013) suggesting that the membrane GR follow an inverted U shape dose response curve as proposed by Prager and Johnson (2009). These findings suggest that either cortisol levels acting on mGR alone or corticosterone levels acting on mGR and mMR rapidly regulate spine levels in a reversible and cyclic manner. Further evidence suggests a synergistic relationship between mGR and mMR for spine increase and normalization respectively (see Figure 1). Like mGR, mMR are also observed at the PSD (Prager et al., 2010) where they are also positioned to interact both with NMDA and AMPA receptors and the ERK/MAPK and LIM kinase pathways to regulate rapid changes in spine number and size. Gan and colleagues observed an MR dependent reversal of spine density; however this effect was long acting, suggesting genomic transcription. Contrary to these findings, Joels and colleagues and Groc and colleagues (and many others) have amassed significant evidence on the rapid non-genomic actions of mMR at synapses. These studies include regulation of post-synaptic AMPA receptors trafficking (Groc et al., 2008) and pre-synaptic regulation of glutamate release (Karst et al., 1999). Cyclic increases and decreases in spine numbers by corticosterone, are likely regulated by synaptic mMR interacting with NMDA and AMPA receptors and the ERK/MAPK and LIM kinase pathways. Thus, low levels of corticosterone will occupy mMR when spines are being returned to their basal levels (see Figure 1 and Prager and Johnson, 2009).

**Figure 1:**
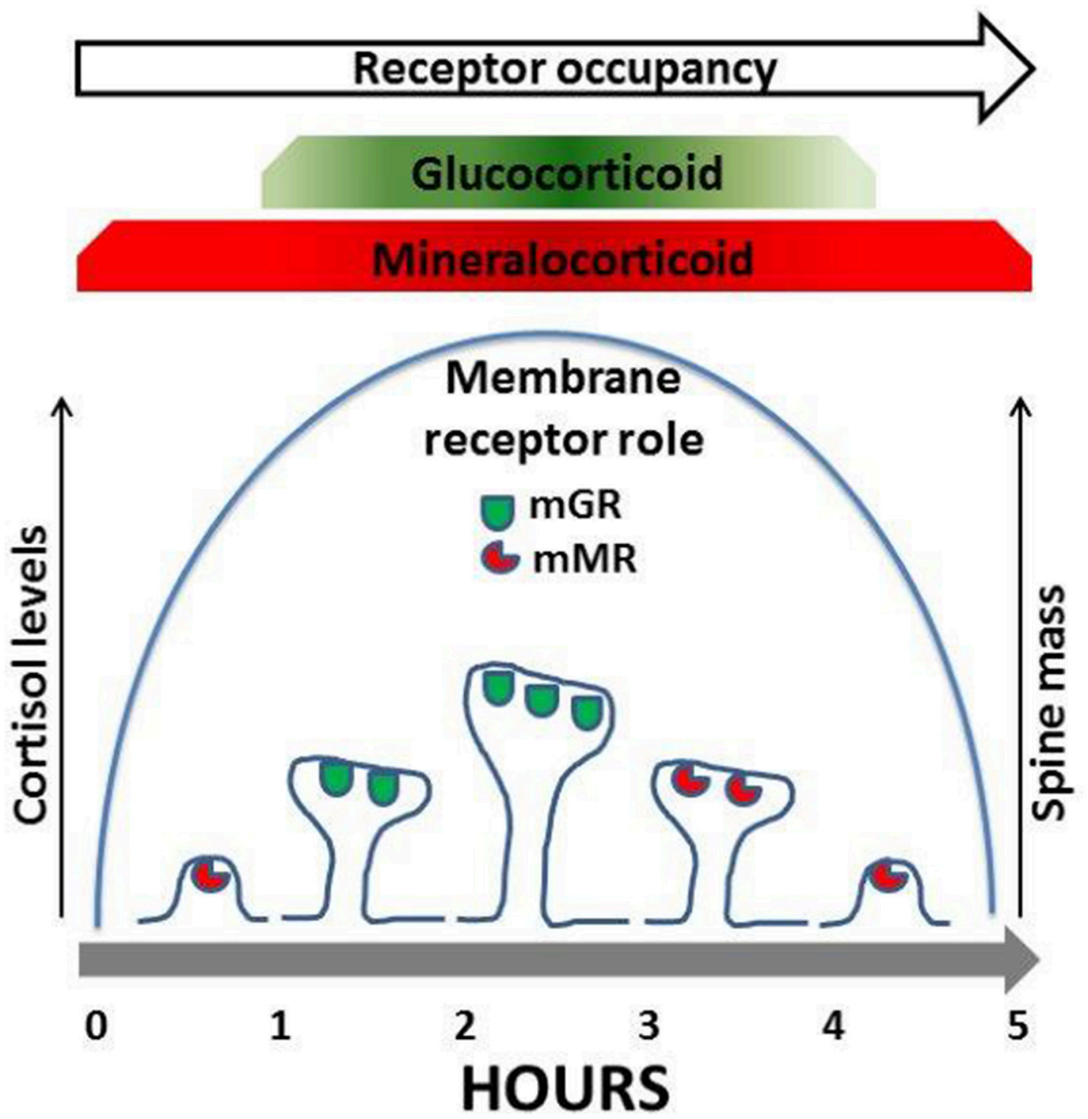
**Membrane MR and GR (mMR, mGR) located at dendritic spines regulate spine mass**. In response to increases in cortisol spines increase in number and size. Mechanistically mGR regulate an increase in dendritic spine num ber and size. In contrast mMR regulates an elimination of dendritic spines. Both mMR and mGR have been identified in the PSO of dendritic spines (see Johnson et al, 2005; Prager and Johnson, 2009; Prager et al, 2010; Yoshiya et al, 2013; and also see Liston et al, 2013).

In summary, thanks to the pioneering work of McEwen and Orchinick and others we are finally at a turning point in our knowledge of the important function of the fast acting membrane form of the mineralocorticoid and glucocorticoid receptors. After 45 years of glucocorticoids in the brain, we are now understanding both the anatomy and the function of fast acting mMR and mGR. A recent and growing body of evidence demonstrate that: GR are located in synapses (Johnson et al., 2005); MR are located in synapses (Prager et al., 2010); GR rapidly regulates synaptic transmission by several mechanisms, including GR regulation of AMPA (Conboy and Sandi, 2010) and GR regulation of pre- and post-synaptic function (Tasker et al., 2006). MR regulates pre-synaptic release (Karst et al., 1999) MR rapidly regulates AMPA receptor membrane insertion (Groc et al., 2008). Furthermore, recent and emerging evidence demonstrates rapid membrane dependent mGR regulation of spine number and size (Komatsuzaki et al., 2012; Liston et al., 2013). Thus, fast acting membrane associated form of the mineralocorticoid and glucocorticoid receptor have multiple roles, with a convergence of functions at synapses and dendrite spines—mMR and mGR at the PSD can trigger circadian and stress induced changes in spine density and shape underlying environmental and endogenous regulation of neural networks involved in memory, learning and other key neurological functions.

## Author contributions

All authors listed, have made substantial, direct and intellectual contribution to the work, and approved it for publication.

### Conflict of interest statement

The authors declare that the research was conducted in the absence of any commercial or financial relationships that could be construed as a potential conflict of interest.
